# Supplementary material for: Polyketide synthase gene clusters in the ericoid mycorrhizal fungus Oidiodendron maius: genome-wide prediction, expression profiling, and functional characterization of knockout mutants
Source: Front Plant Sci. 2026 Sep 16;17:1924430. doi: 10.3389/fpls.2026.1924430 (PMC13623903; doi:10.3389/fpls.2026.1924430)
Supplement: Supplementary file 1 [file SupplementaryFile1.zip › Supplementary Materials/Supplementary Folder 1.zip!/Oidma1_AssemblyScaffolds_Repeatmasked/knownclusterblast/cluster44/jgi.p_Oidma1_207236_mibig_hits.html]

| MIBiG Protein | Description | MIBiG Cluster | MiBiG Product | % ID | % Coverage | BLAST Score | E-value |
| --- | --- | --- | --- | --- | --- | --- | --- |
| ADM79461.1 | O-methyltransferase | BGC0001266 | Grayanic\_acid\_biosynthetic\_gene\_cluster | 34 | 97.8922716628 | 221 | 1.7e-57 |
| ABC79591.2 | cercosporin\_toxin\_biosynthesis\_protein | BGC0001247\_c2 | Cercosporin\_biosynthetic\_gene\_cluster | 31 | 94.6135831382 | 195 | 1.7e-49 |
| CCE67071.1 | methyltransferase | BGC0001242 | Fusarubin\_biosynthetic\_gene\_cluster | 29 | 89.9297423888 | 176 | 8.3e-44 |
| OAQ83753.1 | sterigmatocystin\_8-O-methyltransferase | BGC0001358 | Leucinostatins\_biosynthetic\_gene\_cluster | 28 | 101.639344262 | 145 | 1.2e-34 |
| BAH23997.1 | O-methyltransferase | BGC0000356 | Fumitremorgin\_biosynthetic\_gene\_cluster | 28 | 80.5620608899 | 135 | 1.6e-31 |
| AAS90041.1 | OmtA | BGC0000008 | Aflatoxin\_biosynthetic\_gene\_cluster | 33 | 71.662763466 | 133 | 6.2e-31 |
| BAE71329.1 | o-methyltransferase\_A | BGC0000004 | Aflatoxin\_biosynthetic\_gene\_cluster | 33 | 71.662763466 | 133 | 6.2e-31 |
| AAS90065.1 | OmtA | BGC0000009 | Aflatoxin\_biosynthetic\_gene\_cluster | 29 | 100.234192037 | 133 | 8.1e-31 |
| AAS90087.1 | OmtA | BGC0000010 | Aflatoxin\_biosynthetic\_gene\_cluster | 33 | 71.662763466 | 132 | 1.4e-30 |
| AKD43502.1 | O-Methyltransferase | BGC0001409 | Dutomycin\_biosynthetic\_gene\_cluster | 30 | 71.662763466 | 98 | 2.2e-20 |
| AAQ08925.1 | putative\_o-methyltransferase | BGC0000224 | Fredericamycin\_biosynthetic\_gene\_cluster | 27 | 69.5550351288 | 93 | 7.1e-19 |
| ACN64847.1 | PokMT3 | BGC0001061 | Polyketomycin\_biosynthetic\_gene\_cluster | 29 | 71.4285714286 | 89 | 7.8e-18 |
| CAF31570.1 | putative\_O-methyltransferase | BGC0000705 | Kanamycin\_biosynthetic\_gene\_cluster | 27 | 67.681498829 | 83 | 5.6e-16 |
| CAF60515.1 | putative\_O-methyltransferase | BGC0000704 | Kanamycin\_biosynthetic\_gene\_cluster | 27 | 67.681498829 | 83 | 5.6e-16 |
| BAE95598.1 | putative\_O-methyltransferase | BGC0000703 | Kanamycin\_biosynthetic\_gene\_cluster | 27 | 67.681498829 | 83 | 5.6e-16 |
| ADI24956.1 | GsfD | BGC0000070 | Griseofulvin\_biosynthetic\_gene\_cluster | 26 | 75.1756440281 | 83 | 5.6e-16 |
| ADI24955.1 | GsfC | BGC0000070 | Griseofulvin\_biosynthetic\_gene\_cluster | 25 | 88.5245901639 | 79 | 1.1e-14 |
| EAL85127.1 | O-methyltransferase,\_putative | BGC0001067 | Fumagillin\_biosynthetic\_gene\_cluster | 26 | 50.5854800937 | 73 | 5.8e-13 |
| CAE17534.1 | O-methyltransferase | BGC0000210 | Chromomycin\_A3\_biosynthetic\_gene\_cluster | 25 | 47.775175644 | 65 | 1.6e-10 |
| AAM33664.1 | putative\_methyltransferase\_GrhL | BGC0000230 | Griseorhodin\_biosynthetic\_gene\_cluster | 24 | 61.1241217799 | 58 | 2.5e-08 |
